# Digestive Stability and Bioaccessibility of Antioxidants in Prickly Pear Fruits from the Canary Islands: Healthy Foods and Ingredients

**DOI:** 10.3390/antiox9020164

**Published:** 2020-02-17

**Authors:** Andrea Gómez-Maqueo, Marilena Antunes-Ricardo, Jorge Welti-Chanes, M. Pilar Cano

**Affiliations:** 1 Laboratory of Phytochemistry and Plant Food Functionality, Biotechnology and Food Microbiology Department, Institute of Food Science Research (CIAL) (CSIC-UAM), Nicolás Cabrera 9, 28049 Madrid, Spain; agmaqueo@gmail.com; 2 Tecnologico de Monterrey, Centro de Biotecnología FEMSA, Escuela de Ingeniería y Ciencias, Eugenio Garza Sada 2501, 64700 Monterrey NL, Mexico; marilena.antunes@tec.mx (M.A.-R.); jwelti@tec.mx (J.W.-C.)

**Keywords:** *Opuntia fics-indica*, betalains, phenolic compounds, isorhamnetin glycosides, gastrointestinal digestion, INFOGEST^®^, cactus

## Abstract

Although prickly pear fruits have become an important part of the Canary diet, their native varieties are yet to be characterized in terms of betalains and phenolic compounds. To exert potential health benefits, these antioxidants must be released from the food matrix and be stable in the gastrointestinal tract. Our aim was to characterize the betalain and phenolic profile of four prickly pear varieties from the Canary Islands (Spain) and determine their digestive stability and bioaccessibility via in vitro gastrointestinal digestion. Digestive studies were performed considering the (i) importance of the edible fraction (pulps) and (ii) potential of fruit peels as by-products to obtain healthy ingredients. Betalains and phenolic profiles were analyzed by HPLC-DAD-ESI/MS and HPLC-DAD-MS/QTOF. Pulps in Colorada and Fresa varieties presented high indicaxanthin and betanin content, respectively. Despite low pH in the gastric phase, betalains were stable to reach the intestinal phase, although indicaxanthin presented a higher bioaccessibility. Blanco Buenavista peels contained a distinct flavonoid profile including a new isorhamnetin-hexosyl-rhamnoside. Phenolic compounds were abundant and highly bioaccessible in fruit peels. These findings suggest that prickly pear pulps are rich in bioaccessible betalains; and that their peels could be proposed as potential by-products to obtain sustainable healthy ingredients.

## 1. Introduction

In Spain, prickly pears (*Opuntia ficus-indica* L. Mill.) were introduced after the discovery of America and their expansion was favored by their capability of spreading into large clonal colonies with low water requirements. Nowadays, both wild and cultivated prickly pears may be found growing in peninsular Spain as well as on the Canary Islands. In the island of Tenerife, prickly pear fruits were essential during droughts because they were one of the few crops that could be preserved in good state. Nowadays, prickly pear fruits have become part of the canary diet and are of special interest to catering and hostelry industries. Over 15 million tourists visited the Canary Islands in 2018 [1] most of European origin, to whom the relationship between food and health is very relevant.

Prickly pears are seasonal fruits and can only be obtained for a short period of time throughout the year, typically from June to September (northern hemisphere). However, on the Canary Islands, they may still be available up to late December. The four prickly pear varieties included in this study are the most relevant in Tenerife island. Besides consumed fresh, these may be processed into derived products such as juices and beverages so they may be available all year round. 

Because prickly pears are interesting low-cost sources of healthy foods, their antioxidants have been extensively studied. Several studies reported their total betalains and phenolic compounds using spectrophotometric methods [2,3]. Others have focused on the characterization of betalains and phenolic compounds in prickly pears by high-performance liquid chromatography [4,5,6]. However, because of their lack of commercial standards, only a handful of authors have identified and quantified these compounds in prickly pear fruits using advanced chromatography techniques [7,8,9].

Because of their antioxidants content, prickly pear fruits have been associated with numerous health benefits. Red and purple colored varieties contain betanin, associated with in vitro anti-inflammatory activity, hepatic protective functions, and modulation of gene expression [10,11,12,13]. Yellow and orange colored varieties are rich in indicaxanthin, which gains specific access to selected brain areas and modulates the bioelectric activity of neurons in vivo [14,15]. Prickly pears are also sources of piscidic acid (phenolic acid) and isorhamnetin (flavonoid) mainly found as glycosides. Piscidic acid and isorhamnetin glycosides have shown anti-hypercholesterolemia effects by inhibiting cholesterol permeation in vitro [16] and have been identified in *O. ficus-indica* extracts with anti-inflammatory activity [17,18]. Additionally, the in vitro antioxidant, anti-inflammatory, and anti-hyperglycemic activities of isolated, purified, and semi-synthesized standards from prickly pear fruits of phenolic and betalainic nature have been recently reported by our group [19]. 

However, for antioxidants to exert mentioned health benefits, they should be previously released or decompartmentalized from the cellular structures in which they are contained upon ingestion (mastication) and during gastrointestinal digestion. Antioxidants should remain stable during the gastrointestinal tract so they can be absorbed by our bodies. Bioaccessibility refers to the quantity of a compound that is released from the food matrix in the gastrointestinal tract and is available for absorption. Digestive stability and bioaccessibility can be assessed in vitro via simulated gastrointestinal digestion protocols. The protocol used in this study is an international consensus for the gastrointestinal digestion simulation of foods from the INFOGEST^®^ network [20]. Although the digestive stability and bioaccessibility of betalains in red and yellow Sicilian prickly pear pulps has been previously reported [21], we are interested in analyzing the digestive stability of betalains and phenolic compounds in pulps and peels of red, orange, and white prickly pear varieties from the Canary Islands. Peels were equally assessed in this study as a sustainable proposal to reduce agri-food industry wastes and obtain relevant and inexpensive sources of antioxidants. 

The aim of this work was to characterize and quantify betalains and phenolic compounds in four prickly pear varieties from the Canary Islands (Spain) and study their digestive stability and bioaccessibility via in vitro gastrointestinal digestion according to the INFOGEST^®^ consensus. Digestive stability of antioxidants in pulps and peels were evaluated (i) because of the of the relevance of the edible fraction (pulp) and (ii) to explore the possibility of using peels as potential healthy ingredients.

## 2. Materials and Methods

### 2.1. Solvents, Reagents, and Standards

Methanol (99.8% LC-MS) was purchased from VWR International (Barcelona, Spain). Ultra-pure water was obtained from a Milipak^®^ Express 40 system (Merk-Milipore, Dormstadt, Germany). Formic acid was purchased from Panreac Química (Barcelona, Spain). Sephadex LH-20, standards (isorhamnetin, quercetin, rutin, gallic acid, 4-hydroxybenzoic acid), amino acids (glycine, asparagine, glutamine, glutamic acid, proline, and tryptophan), α-amylase (10080; 79 U mg/L), pepsin (P6887; 791 U mg/L), pancreatin (P7545, 17 units TAME per mg), bile (B8381), and other reagents used for the in vitro digestion assay were purchased from Sigma-Aldrich (St. Louis, MO, USA).

Betanin was purified from a betalain-rich concentrate extracted from commercial beetroot and betaxanthins were semi-synthesized using purified betanin [8]. Piscidic acid was purified from prickly pear peels by semi-preparative high-performance liquid chromatography (HPLC) [8]. Standards for isorhamnetin and kaempherol glycosides were kindly provided by Dr. Serna-Saldivar’s laboratory from Centro de Biotecnologia FEMSA (Escuela de Ingeniería y Ciencias, Instituto Tecnológico de Monterrey, Monterrey, Mexico), where these compounds were previously isolated from *Opuntia* cladodes [22,23]. All isolated and semi-synthetized standards were analyzed for authenticity and purity by HPLC-ESI-MS-QTof.

### 2.2. Prickly Pears and Physicochemical Analysis

Orange Colorada, red Fresa, and white Blanco Fasnia prickly pear *(Opuntia ficus-indica* L. Mill.) varieties were obtained from Fasnia (Tenerife, Canary Islands, Spain; 28°2′ N, 16°4′ W; 446 m over sea level). White Blanco Buenavista prickly pears *(Opuntia ficus-indica* L. Mill.) were obtained from Buenavista del Norte (Tenerife, Canary Islands, Spain; 28°2′ N, 16°5′ W; 127 m over sea level). Fruits were washed and selected according to uniform maturity, size, and no defects. Their physicochemical characteristics such as apical caliber (cm), equatorial caliber (cm), weight (g), peel, pulp, and seed proportion (%) were determined directly in ten whole fruits of each variety (Table 1). 

Titratable acidity (g citric acid/100 g fresh weight) was determined by neutralization of prickly pear juice with 0.1 N sodium hydroxide until a pH value of 8.1. pH and soluble solids (°Brix at 25 °C) were also measured from juice obtained from prickly pear pulps. Firmness of the fruit was determined by penetration (5 mm) on the fruit (epidermis removed) using a texture analyzer (TA.XT plus texture analyzer, Stable Micro Systems, Godalming, UK). Color of peels and pulps was recorded using the L* (lightness), a* (green-red tonality), and b* (blue-yellow tonality) scale CIELAB system with a Konica Minolta CM-3500d (Japan).

After their physicochemical analysis, prickly pears were separated into peels and pulps. Tissues were cut into small pieces (20 × 20 mm), vacuum-sealed in polyethylene bags, and frozen with liquid nitrogen. Prickly pear tissues were freeze-dried for 5 days at −45 °C and 1.3 × 10^−3^ MPa (LyoBeta 15, Azbil Telstar, S.L., Terrasa, Spain). Samples were pulverized (Grindomix GM200, Retsch, Germany) to a fine particle size (<2 mm) and seeds were removed. Samples were vacuum-sealed and stored at −20 °C until analysis. 

### 2.3. Prickly Pear Extract Obtention for Characterization

For characterization of betalains and phenolic compounds, prickly pear extracts were obtained from freeze-dried and pulverized tissues under diminished light [8]. One gram of freeze-dried prickly pear tissue was extracted with 5 mL methanol:water (1:1, v:v) by homogenizing with a vortex for 1 min and placing for 4 min in an ultrasonic water bath (3000514, 50/60 Hz, 360 W, J.P Selecta S.A., Barcelona, Spain) with ice. Samples were centrifuged for 10 min at 10,000× *g* at 4 °C. The supernatants were collected, and the extraction process was repeated two more times by adding 3 mL of methanol:water (1:1, v:v). Samples were extracted one last time with 3 mL of pure methanol and the combined supernatants were evaporated in a rotary evaporator (Buchi, Flawil, Switzerland) at 30°C to reduce their volume. Aqueous extracts were then made up to 5 mL with ultrapure water, filtered through a 0.45 µm nylon filter (E0032, Análisis Vínicos, Spain), and analyzed by HPLC. 

### 2.4. In Vitro Digestion Assay 

The in vitro digestion assay was performed according to the standardized INFOGEST protocol [20,24] using rehydrated freeze-dried samples. The solutions for mouth (Simulated Saliva Fluid, SSF), stomach (Simulated Gastric Fluid, SGF), and small intestinal (Simulated Duodenal Fluid, SDF) compartments were prepared according to a previous article [25]. The addition of enzymes in the preparation of digestive fluids was performed daily and moments prior to the digestive assay. After each phase (oral, gastric, and intestinal) of the simulated digestion, samples were frozen with liquid N_2_ and stored at −20°.

After the obtaining of all phases of the in vitro gastrointestinal digestion, digestive phases were thawed and extracts containing the betalains and phenolic compounds were obtained [26]. Ten grams of oral phase and 20 g of gastric and intestinal phases were weighed in 50 mL tubes. The pH was set to pH 4 with 0.4 M NaOH or 1 M HCl. Afterwards, a pure methanol solution was added in a 1:1 (v:v) proportion to each tube to precipitate remaining enzymes. Samples were homogenized at 700× *g* for 2 min using an ultrahomogenizer (Omnimixer ES-207, Omni International Inc, Gainsville, FL, USA). Then, they were centrifuged at 4 °C for 15 min at 9000× *g* (Eppendorf Centrifuge 5804 R, Eppendorf ^®^, Eppendorf Ibérica S.L.U, Madrid, Spain) and the supernatant was recovered. Methanol was eliminated from the supernatants using a rotary evaporator at 30 mbar for 30 min. Aqueous samples were filtered with a 0.45 µm syringe filter into a vial and analyzed by HPLC.

The bioaccessibility of antioxidants such as betalains and phenolic compounds were calculated as the ratio between their concentration in the intestinal fraction and their initial concentration in the fruit (Equation (1)).

(1) $$Bioaccessibility\;\left(\%\right)=\frac{Antioxidant\;Compounds\;\mathrm{intestinal}\;\mathrm{phase}}{Antioxidant\;Compounds\;\mathrm{fruit}\;\mathrm{tissue}}\times 100$$

### 2.5. Betalain and Phenolic Content by High Performance Liquid Chromatography

Betalains and phenolic compounds were determined simultaneously by high-performance liquid chromatography [8]. A 1200 Series Agilent HPLC System (Agilent Technologies, Santa Clara, CA, USA) with a reverse-phase C18 column (Zorbax SB-C18, 250 × 4.6 mm i.d., S-5 µm; Agilent) at 25 °C was used. Mobile phase A was 1% formic acid (*v*/*v*) in ultrapure water and mobile phase B was 1% formic acid (*v*/*v*) in methanol. Separation was achieved using an initial composition of 15% B during 15 min, increased to 25% within 10 min, subsequentially ramped to 50% B within 10 min, increased to 75% B in 15 min, and finally followed by a decrease period of 15% B in 5 min prior to isocratic re-equilibration for 10 min. The flow rate was 0.8 mL/min and the injection volume was 20 µL. The UV-visible photodiode array detector was set at four wavelengths to detect phenolic acids (280 nm), flavonoids (370 nm), betaxanthins (480 nm), and betacyanins (535 nm). UV/Vis spectra were additionally recorded between 200 and 700 nm. The HPLC-DAD was coupled to a mass spectrometry detector (LCMS SQ 6120, Agilent, Agilent Technologies, Santa Clara, California, USA) with an electrospray ionization (ESI) source operating in positive ion mode. The drying gas was nitrogen at 3 L/min at 137.9 KPa. The nebulizer temperature was 300 °C and the capillary had 3500 V potential. The coliseum gas was helium and the fragmentation amplitude were 70 V. Spectra were recorded m/z from 100 to 1000. 

Further mass spectrometry analyses were performed in a maXis II LC-QTOF equipment (Bruker Daltonics, Bremen, Germany) with an ESI source and the same chromatographic conditions. The ESI-QTOF detector worked in positive ion mode and recorded spectra *m*/*z* from 50 to 3000. Operation conditions were 300 °C, capillary voltage 3500 V, charging voltage 2000 V, nebulizer 2.0 bar, and dry gas at 6 L/min. MS/MS analysis used the bbCID (Broad Band Collision Induces Dissociation) method at 30 eV.

Compounds were identified according to their retention times, UV/Vis, and mass spectral data compared to those of commercial, semi-synthesized, or purified standards. Identification and quantitation of portulacaxanthin, vulgaxanthin I and II, indicaxanthin, betanin, betanidin, piscidic acid, isorhamnetin glycosides (IG1, IG2, IG3, IG4, IG5, and IG7), kampferol glycoside (KG1), and rutin were determined using standards and their respective calibration curves. Quercetin glycosides were quantified by using the rutin calibration curve. Remaining betalains were quantified using the calibration curves of betalains of similar molecular weight. 4-hydroxybenzoic acid derivative was quantified using the calibration curve for 4-hydroxybenzoic acid. The identification of betalains and phenolic compounds in prickly pears is presented in Table 2.

### 2.6. Statistical Analysis

Results were expressed as mean ± standard deviation (*n* = 4). This came from obtaining at least two independent extracts or digestive phases (*n* = 2) and by performing the determinations of each two times (*n* = 2). Significant differences (*p* < 0.5) were calculated by one-way analysis of variance (ANOVA), followed by a post hoc Tukey’s test. Statistical analyses were performed with IBM^®^ SPSS^®^ Statistics 23.0 (IBM Corp, Armonk, NY, USA).

## 3. Results and Discussion

### 3.1. Physicochemical Characteristics

The physical appearance and physicochemical characteristics of prickly pears from the Canary Islands is presented in Table 1. Regarding the colored varieties, Colorada was characteristic for its orange pulp and peel. Although Fresa had fuchsia tonalities, it was classified as a red variety due to its betacyanin/betaxanthin ratio and flavonoid profile (see Section 3.2 and Section 3.3). The white varieties were easily recognizable from one another. Blanco Buenavista was characterized by a green peel (exterior) and white pulp, while Blanco Fasnia presented a yellow/green peel and white pulp. Among varieties, there were no significant differences in terms of size (cm), weight (g), proportion (%) of peel, pulps, and seeds, and titratable acidity. However, the colored varieties (Colorada and Fresa) presented total soluble solids of 13.4% and 13.6%, lower than the white varieties (15.7–16%). Colored varieties were considered ripe between 11 and 14 °Brix, while white prickly pear varieties reached up to 16 °Brix when ripe. 

### 3.2. Identification of Betalains and Phenolic Compounds

**Figure 1:**
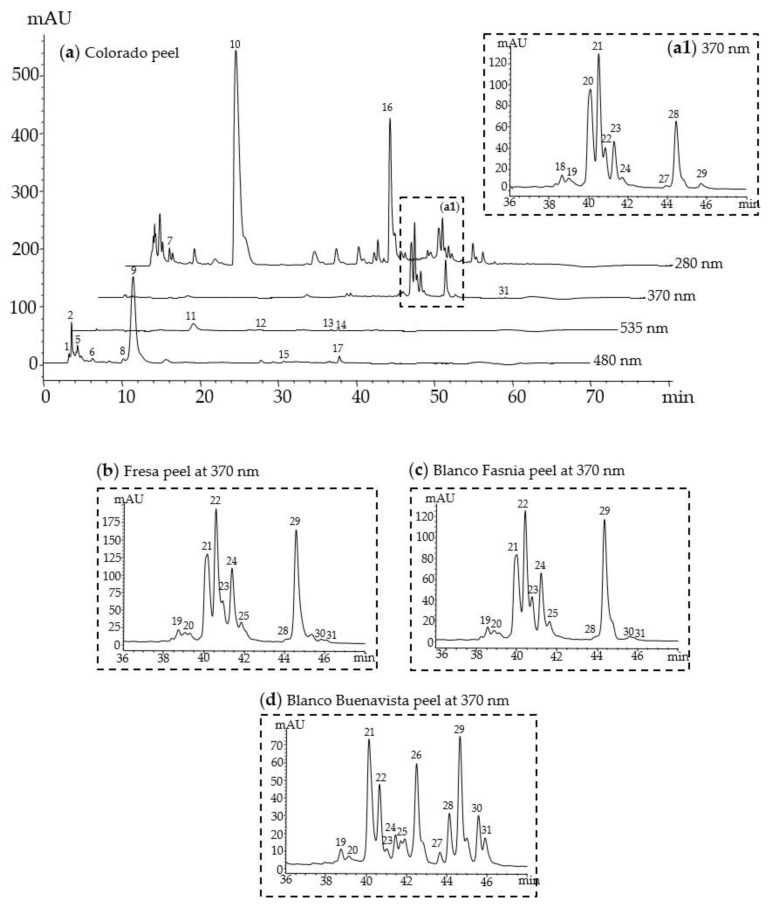
HPLC C18 Chromatogram of betalains and phenolic compounds from (**a**) Colorada prickly pear (*Opuntia ficus-indica* L. Mill.) peel at 280, 370, 480, and 535 nm and (**b**) Fresa peel, (**c**) Blanco Fasnia peel, and (**d**) Blanco Buenavista peel at 370 nm.

The profile of betalains and phenolic compounds was analyzed using HPLC-DAD-ESI/MS (with ESI and QTof detectors) with absorbance detection at 280, 370, 480, and 535 nm, to identify phenolic acids, flavonoids, betaxanthin, and betacyanins, respectively. Figure 1 shows the simultaneous determination of these bioactive compounds. The HPLC retention times, UV/Vis spectra, and MS spectral data of the principal peaks are shown in Table 2.

The HPLC analysis monitored at 480 and 535 nm allowed the identification of 14 betalains (9 betaxanthins and 5 betacyanins). Peak 9 (Rt = 10.5; λ_max_ at 478 nm) was identified as bx-proline (indicaxanthin), the major betaxanthin in orange prickly pears. Peak 11 (Rt = 15.7; λ_max_ at 534 nm) was identified as betanin, the major betacyanin in red prickly pears. The HPLC retention times, UV/Vis spectra, and MS spectral data matched our reference standards.

We identified 17 phenolic compounds, which included 3 phenolic acids (λ_max_ near 280 nm) and 14 flavonoids (λ_max_ near 370 nm). Peak 10 (Rt = 14.0; λ_max_ at 232 and 275 nm) was a common phenolic acid found in cactus called piscidic acid. Another phenolic acid, 4-hydroxybenzoic acid derivative (peak 16; Rt = 33.7; λ_max_ at 274 nm), was also identified. Regarding flavonoids, the most abundant were isorhamnetin glycosides, namely isorhamnetin glucosyl-rhamnosyl-rhamnoside (IG1) (peak 20; Rt = 40.0; λ_max_ at 254 and 354 nm), isorhamnetin glucosyl-rhamnosyl-pentoside (IG2) (peak 21; Rt = 40.4; λ_max_ at 253 and 354 nm), isorhamnetin glucosyl-hexosyl-hexosyl-pentoside (IG3) (peak 22; Rt = 40.8; λ_max_ at 253 and 354 nm), isorhamnetin glucosyl-pentoside (IG4) (peak 23; Rt = 41.2; λ_max_ at 254 and 354 nm), and isorhamnetin glucosyl-rhamnoside (IG5) (peak 28; Rt = 44.5; λ_max_ at 253 and 354 nm) (Figure 1). These flavonoids were first characterized in cladodes [22], while they have also been reported in *O. ficus-indica* fruits or juices [27,28].

**Figure 2:**
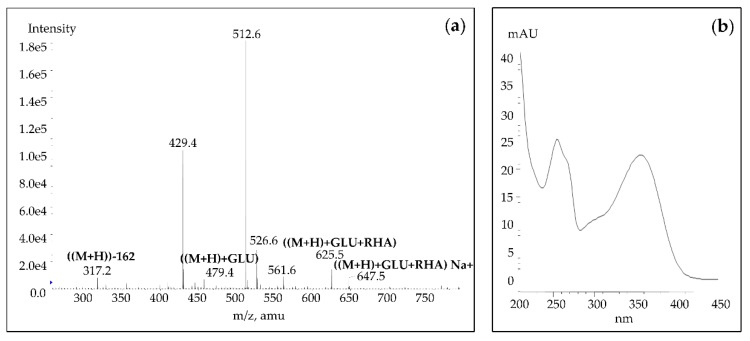
(**a**) Mass spectral data and (**b**) UV/Vis spectra of isorhamnetin hexosyl-rhamnoside (IG7).

In the present study, we found that the white Blanco Buenavista variety contains a more complex flavonoid profile than other varieties. As shown in Figure 1d, it is characterized by a high IG1 and IG5 content, but additionally contains peak 26 (Rt = 42.5; λ_max_ at 255 and 355 nm), which we identified as isorhamnetin hexosyl-rhamnoside for the first time in prickly pear fruits. Following the abbreviation of isorhamnetin glycosides purified from cacti [18,19], we abbreviated this compound as IG7. This last isorhamnetin glycoside could be an isomer of IG5, however more information is needed to elucidate its structure. The mass spectral data and UV/Vis spectra of this last compound are presented in Figure 2. Peak 29 (Rt = 45.5; λ_max_ at 253 and 354 nm) isorhamnetin hexosyl-pentoside (IG6) was also identified in the white variety from Buenavista. This flavonoid was previously identified in cladode extracts from Mexican *Opuntia ficus-indica* (L.) Mill. var. Jalapa and var. Villanueva [22]. 

### 3.3. Quantification of Betalains

Betalain content in prickly pears varieties from the Canary Islands is presented in Table 3. Colored prickly pears var. Colorada and Fresa are rich in betalains, which give them their characteristic color. Betalains were more abundant in pulps than in peels. Despite Colorada’s diverse betaxanthin profile, it was mainly rich in indicaxanthin. Indicaxanthin content in Colorada was 11.79 mg/100 g fresh pulp and 5.98 mg/100 g fresh peel. Indicaxanthin content in pulps was comparable to yellow Sicilian prickly pears (12.32 mg/100 g fresh pulp) [21]. Indicaxanthin is an interesting molecule as suggested by the overview on the therapeutic effects ranging from the anti-inflammatory to the neuro-modulatory and anti-tumoral ones [29]. Furthermore, Colorada also contained significant amounts of other betaxanthins such as portulacaxanthin, vulgaxanthin III, vulgaxanthin I, and vulgaxanthin II, among others. Colorada prickly pears also contained 0.62 mg betanin/100 g fresh pulp 0.54 mg betanin/100 g fresh peel). Compared to yellow Spanish Verdal and yellow Mexican Diamante prickly pear cultivars, orange Spanish Colorada pulp contained 6- and 3-fold as many total betalains [8]. 

The red Fresa variety was mostly rich in red-colored betacyanins such as betanin, isobetanin, betanidin, gomphrenin I, and neobetanin. Betanin content in Fresa prickly pears was 11.17 mg/100 g fresh pulp and 9.54 mg/100 g fresh peel. Interestingly, this red variety also contained 4.01 and 1.24 mg indicaxanthin/100 g fresh pulp and peel, respectively. This betanin/indicaxanthin ratio (11/4) is similar to red Sicilian pricky pears containing 14.1 mg betanin/100 g fresh pulp and 4.70 mg indicaxanthin/100 g fresh pulp) [21]. Compared to red Mexican Liria and Roja Lisa cultivars, red Spanish Fresa prickly pear pulps contain three times more betanin [6]. Red Spanish Sanguinos, red Mexican Vigor, and purple Spanish Morada varieties possess significantly lower betanin content than red Spanish Fresa prickly pears, however the purple Mexican Pelota variety had a higher betanin content [8]. 

White Blanco Buenavista and Blanco Fasnia prickly pears contained 0.11 and 0.14 mg total betalains/100 g fresh weight. Despite their low betalain content, they contained more yellow-colored betaxanthins than red-colored betacyanins. 

### 3.4. Quantification of Phenolic Compounds

The phenolic content in prickly pear pulps and peels is reported in Table 4. Prickly pear pulps contained 38–62 mg total phenolic compounds/100 g fresh pulp, which consisted almost entirely of phenolic acids content, being the most abundant piscidic acid. 

As expected, prickly pear peels had a significantly higher phenolic content than pulps and were also rich in piscidic acid (307–407 mg/100 g fresh peel). Fresa, Colorada, and Blanco Buenavista varieties contained 430–452 mg total phenolic compounds/10g fresh peel. However, Blanco Fasnia was characterized by a lower phenolic content of 327 mg/100 g fresh peel. 

Total flavonoid content in peels was 8-14 mg total flavonoids/100g fresh peel. It has been suggested that this flavonoid profile could serve as a chemical fingerprint regarding genuineness of cactus *O. ficus-indica* fruits [27]. Colorada, Fresa, and Blanco Fasnia presented a high IG1 and IG5 content (Figure 1, Table 4), which is typical for yellow and red prickly pear varieties. Despite Fresa’s magenta-purple color and high betanin content (reported in red and purple varieties), we decided to classify it as a red variety because of its isorhamnetin glycoside profile. As reported in another study, purple prickly pears possess a higher IG1 and IG5 flavonoid profile [8]. Blanco Buenavista peels presented a new flavonoid profile consisting of high IG1, IG5, and a new isorhamnetin hexosyl-rhamnoside, which we abbreviated IG7. 

### 3.5. Digestive Stability of Betalains and Phenolic Compounds 

#### 3.5.1. Digestive Stability in Pulps (Edible Fraction)

**Figure 3:**
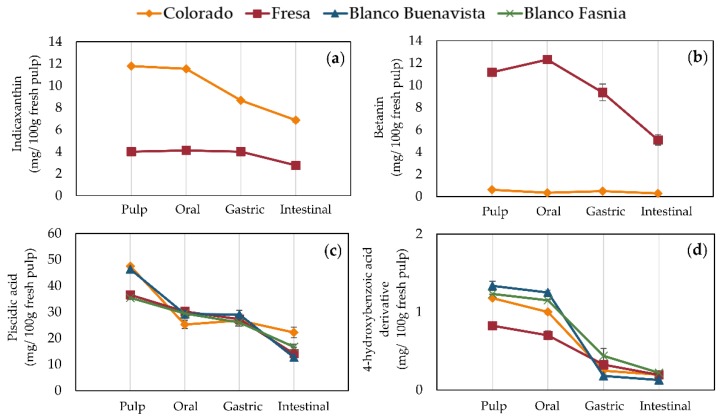
Bioactive content (mg/100 g fresh weight) in prickly pear pulps during in vitro simulated gastrointestinal digestion. (**a**) indicaxanthin, (**b**) betanin, (**c**) piscidic acid, and (**d**) 4-hydroxybenzoic acid derivative.

The digestive stability of antioxidants in prickly pears form the Canary Islands (Spain) is shown in Figure 3 and raw data may be consulted in Supplementary Table S1. Indicaxanthin in Colorada prickly pears underwent a 26% loss during the gastric phase and reached a final loss of 42% in the intestinal phase. In the Fresa variety, indicaxanthin loss did not occur until the intestinal phase where it presented only a 31% loss (Figure 3a). 

Betanin in Fresa prickly pear pulps also decreased 21% in the gastric phase and 55% in the intestinal phase (Figure 3b). These observed betalain losses are comparable to what has been previously reported for red-colored Sicilian prickly pears [21]. Similarly, in red dragon fruit drink and pressed red dragon fruit juice, betanin stability during simulated gastrointestinal digestion suffered a minor loss (<25%) in a gastric-like environment, and greater loss during the intestinal phase [30]. Betalains are only stable at pH from 3–7, which explains their decay in the gastric phase.

During the in vitro gastrointestinal digestion, piscidic acid from all prickly pear varieties reached a similar concentration in the oral phase (Figure 3c) ranging from 25 to 30 mg/100 g fresh pulp and was further degraded in the intestinal phase. The final concentrations of piscidic acid in the intestinal phase of digested Colorada, Fresa, Blanco Buenavista, and Blanco Fasnia were 22.19, 14.08, 12.70, and 16.68 mg piscidic acid/100 g fresh pulp, respectively, which corresponded to losses of 53–71%. In other terms, 4-hydroxybenzoic acid derivative (Figure 3d) was greatly lost in the gastric phase and was the least stable of the evaluated antioxidants in prickly pear pulps when submitted to in vitro gastrointestinal digestion.

#### 3.5.2. Digestive Stability in Peels (by-Products to Obtain Potential Healthy Ingredients)

**Figure 4:**
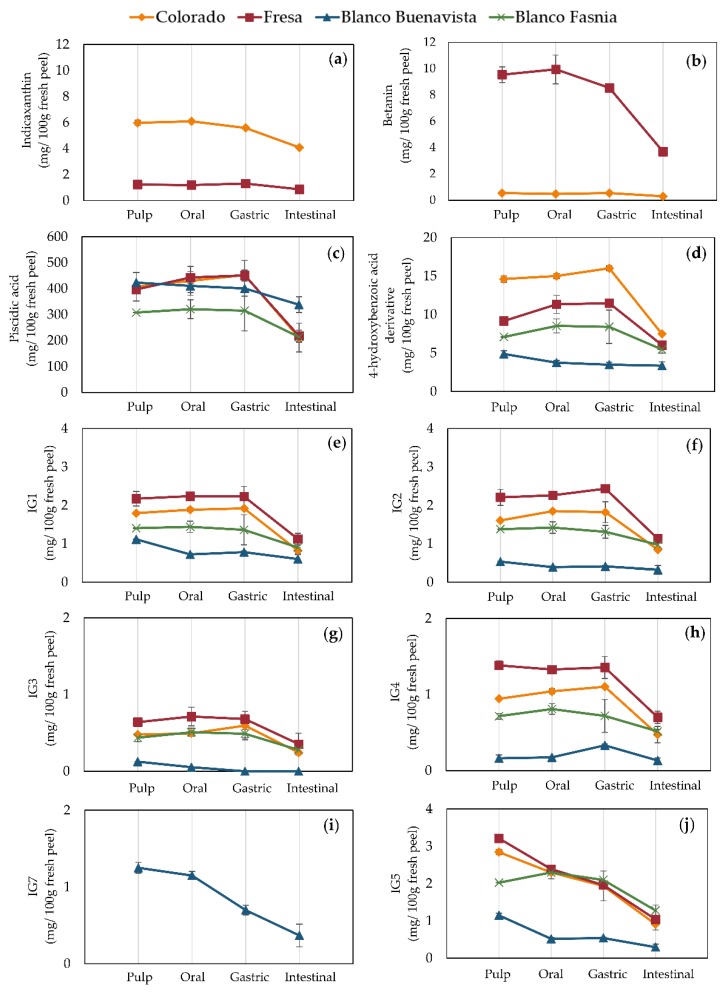
Bioactive content (mg/100 g fresh weight) in prickly pear peels during in vitro simulated gastrointestinal digestion. (**a**) indicaxanthin, (**b**) betanin, (**c**) piscidic acid, and (**d**) 4-hydroxybenzoic acid derivative, (e) isorhamnetin glucosyl-rhamnosyl-rhamnoside (IG1), (**f**) isorhamnetin glucosyl-rhamnosyl-pentoside (IG2), (**g**) isorhamnetin hexosyl-hexosyl-pentoside (IG3), (**h**) isorhamnetin glucosyl-pentoside (IG4), (**i**) isorhamnetin hexosyl-rhamnoside (IG7), (**j**) isorhamnetin glucosyl-rhamnoside (IG5).

The digestive stability of antioxidants in prickly pear peels from the Canary Islands is shown in Figure 4 and raw data may be consulted in Supplementary Table S2. In the gastric phase, betanin and indicaxanthin were more stable in peel tissue than in pulp tissue (Figure 4a,b). However, in the intestinal phase, its recovery was similar. In the intestinal phase of digested Fresa peels, betanin loss was of 54%. Although present in less concentration, betanin loss in the intestinal phase of Colorada prickly pear peels was 46%. These results are similar to the data reported for pure betanin in an in vivo simulated gastrointestinal digestion and ex vivo colonic fermentation [31]. The mentioned study showed 7% loss in the oral fluid, 36% loss in the gastric fluid, 46% loss in the small intestine fluid, and was not detected after the colonic fermentation. Despite differences in indicaxanthin deposition in prickly pear peels (stored in vesicles in the cytoplasm) and pulps (stored in vacuoles) [32], indicaxanthin digestive stability was similar in both tissues.

Phenolic acids in digested prickly pear peels were stable throughout the oral and gastric phases, only decreasing until the intestinal phase (Figure 4c,d). In the intestinal phase of digested peels, piscidic acid showed lower losses (20–45%) compared to pulps (53–71%). 4-hydroxybenzoic acid derivative in the intestinal phase was also more stable in peels, presenting 23–49% loss compared to 77–90% loss in pulps. This may be due to the composition of prickly pear peels, which act as a protection from the digestive milieu.

Isorhamnetin glycosides IG1, IG2, IG3, and IG4 were also stable during the gastric phase and only degraded in the intestinal phase (Figure 4g–j). Their recoveries in the intestinal phase were of 46–64%, 52–70%, 50–64%, and 49–81%, respectively. In other terms, IG5 and IG7 were affected when passing through the oral, gastric, and intestinal phases. The only comparative recovery in the intestinal phase of IG5 with the other isorhamnetin glycosides was in Blanco Fasnia of 63%. The other varieties (Colorado, Fresa, and Blanco Buenavista) showed lower recoveries of IG5 (26–32%) and IG7 (30%). The stability of phenolic compounds during digestion partially depends on its glycosylation pattern; for instance, isorhamnetin glycosides from *Opuntia ficus-indica* cladodes are more bioaccessible than its respective aglycones [18]. It has been reported that after intravenous dose of the isorhamnetin standard, the elimination half-life was 0.64 h but increased to 1.08 h when the *O. ficus-indica* extract was administered suggesting that isorhamnetin glycosides naturally found in *O. ficus-indica* cladodes could be a controlled delivery system to maintain a constant plasmatic concentration of this important flavonoid to exert its biological effects in vivo.

### 3.6. Bioaccessiblity of Betalains and Phenolic Compounds

#### 3.6.1. Bioaccessibility in Pulps (Edible Fraction)

The bioaccessibility of betalains and phenolic compounds in prickly pear pulps after in vitro gastrointestinal digestion is presented in Table 5. Indicaxanthin was more bioaccessible (52–69%) than betanin with no significant differences between Colorado and Fresa varieties. Betanin bioaccessibility was 46%, comparable to red dragon fruit drink and pressed red dragon fruit juice, which was of 46.4% and 43.8%, respectively [30]. Theoretically, betalains reach the intestinal phase and are absorbed in the epithelial cells of the intestine [33,34,35]. Further studies are required to determine betanin and indicaxanthin absorption in prickly pear varieties from the Canary Islands (Spain). This is of importance because betalains may affect age-related diseases because of their influence on human cell lifespan and aspects of age-dependent atherosclerosis [36,37]. Furthermore, no metabolites or degraded products could be detected by HPLC during the stages of the in vitro gastrointestinal digestion in the present study. This agrees with other studies, which have shown that betanin and indicaxanthin are not metabolized during digestion or in the liver [33,38]. As a further advantage, in vivo studies have shown that betalain bioavailability in prickly pears is higher than in other food matrices such as beetroot [39,40]. 

Regarding bioaccessibility of phenolic compounds in pulps, piscidic acid in Colorado (46.7%), Fresa (38.6%), and Blanco Fasnia (47.1%) was more bioaccessible than in Blanco Buenavista (27.4%). On the other hand, 4-hydoxybenzoic acid derivative was the least bioaccessible compound in this study, presenting statistically significant differences between the four varieties.

#### 3.6.2. Bioaccessibility in Peels (by-Products to Obtain Potential Healthy Ingredients)

The bioaccessibility of betalains and phenolic compounds in prickly pear pulps and peels after in vitro gastrointestinal digestion is shown in Table 5 and Table 6. Betalains, although present at lower concentrations in peels, were equally bioaccessible as in pulps.

The bioaccessibilities of piscidic acid in Colorado (51.8%), Fresa (54.8%), Blanco Buenavista (79.9%), and Blanco Fasnia (69.4%) was high. Further studies on the bioavailability of this phenolic acid are relevant because of its presence in *Opuntia ficus-indica* cladode extracts, which recently have been reported to affect cholesterol levels in vitro by reducing the membrane cholesterol transporter proteins [41]. Compared to pulps, bioaccessibility of 4-hydroxybenzoic acid derivative in prickly pear peels was significantly higher in Fresa (65.8%), Blanco Buenavista (68.9%), and Blanco Fasnia (77.1%). 

White Blanco Buenavista and Blanco Fasnia presented higher bioaccessibility of phenolic compounds in peels than the colored Colorado and Fresa varieties. IG1, IG2, IG3, and IG4 also showed high bioaccessibility. These results in peels bear close similarity to what has been reported for *O. ficus-indica* cladodes [42]. Isorhamentin glycosides in cladodes showed bioaccessibilities of 58%, 38%, and 112% for IG1, IG4, and piscidic acid [43]. These authors also reported that throughout colonic fermentation, flavonoids showed more degradation than phenolic acids and reduced H_2_O_2_-induced DNA damage in HT29 cells [43]. It has also been suggested that flavonoid glycosides have a higher bioaccessibility than their corresponding aglycones due to their improved aqueous solubility, higher stability during digestion, and higher ability to pass across lipid rich biological membranes [18,44]. 

## 4. Conclusions

In this study, we characterized the betalain and phenolic profile of prickly pear fruits (*Opuntia ficus-indica* L. Mill.) from the Canary Islands by HPLC-DAD-MS/QTOF. Additionally, using a standardized in vitro gastrointestinal methodology (INFOGEST^®^), we determined the digestive stability and bioaccessibility of their most relevant antioxidants. Digestive studies were performed in the pulps because of they are the edible fraction of the fruits, and in the peels because they are considered potential by-products to obtain healthy ingredients. We reported a total of 31 antioxidants, which were composed of 3 phenolic acids, 14 flavonoids, 9 betaxanthins, and 5 betacyanins. We found that betalains betanin and indicaxanthin were more abundant in the pulps of colored prickly pear varieties such as Colorado and Fresa where they presented a high bioaccessibility. Prickly pear peels were significantly richer in phenolic compounds such as piscidic acid, 4-hydroxybenzoic acid derivative, and different isorhamnetin glycosides, which had high digestive stability and bioaccessibility. We also identified, for the first time, a new isorhamnetin-hexosyl-rhamnoside and distinct flavonoid profile in the white Blanco Buenavista variety.

In conclusion, prickly pear fruits from the Canary Islands (Spain) represent an interesting fruit for a healthy diet and a promising source of healthy ingredients. On one hand, pulps of their colored varieties are interesting sources of bioaccessible betalains. On the other hand, peels of all studied prickly pear varieties could be utilized as a sustainable source of ingredients with health potential due to their highly bioaccessible phenolic profile.

## Figures and Tables

**Table 1:** Physicochemical characteristics of prickly pears (*Opuntia ficus-indica* L. Mill.) from Canary Islands.

	Prickly Pear (*Opuntia ficus-indica* L. Mill.) Variety			
Characteristics	Colorada *(Orange)*	Fresa *(Red)*	Blanco Buenavista *(White)*	Blanco Fasnia *(White)*
	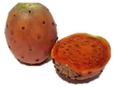	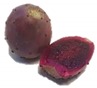	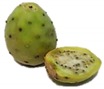	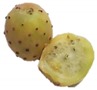
Pulp color	Orange	Fuchsia	White	White
Peel color	Orange	Fuchsia	Green	Yellow/green
Apical caliber (cm)	6.2 ± 0.8 ^a^	5.8 ± 0.5 ^a^	6.6 ± 0.7 ^a^	6.3 ± 1.0 ^a^
Equatorial caliber (cm)	4.9 ± 0.3 ^a^	4.5 ± 0.4 ^a^	5.1 ± 0.3 ^a^	4.6 ± 0.3 ^a^
Weight (g)	106.0 ± 9.3 ^a^	78.9 ± 9.6 ^a^	129.6 ± 23.9 ^a^	89.0 ± 7.0 ^a^
% peel	55.7 ± 2.6 ^a^	41.3 ± 11.4 ^a^	55.4 ± 1.8 ^a^	44.1 ± 4.1 ^a^
% pulp	38.2 ± 3.7 ^a^	51.8 ± 6.6 ^a^	41.1 ± 6.2 ^a^	50.6 ± 4.2 ^a^
% seeds	6.1 ± 0.1 ^a^	6.9 ± 1.4 ^a^	3.5 ± 0.9 ^a^	5.3 ± 0.8 ^a^
pH	6.1 ± 0.2 ^a^	6.1 ± 0.0 ^a^	6.6 ± 0.1 ^b^	6.2 ± 0.1 ^a^
Titratable acidity (%)	0.01 ± 0.00 ^a^	0.01 ± 0.00 ^a^	0.01 ± 0.00 ^a^	0.01 ± 0.00 ^a^
Soluble solids (°Brix)	13.4 ± 0.1 ^a^	13.6 ± 0.1 ^a^	15.7 ± 0.1 ^b^	16.0 ± 0.2 ^b^
Firmness (N)	18.3 ± 3.6 ^a^	12.5 ± 3.8 ^a^	22.0 ± 2.6 ^a^	10.7 ± 1.6 ^a^
Color peel (CIELAB)				
L*	50.4 ± 1.8 ^ab^	41.7 ± 2.4 ^a^	58.9 ± 2.7 ^b^	60.6 ± 3.5 ^b^
a*	9.2 ± 2.4 ^ab^	19.4 ± 3.7 ^b^	1.1 ± 3.6 ^a^	4.3 ± 2.4 ^ab^
b*	11.0 ± 2.3 ^ab^	−4.2 ± 2.2 ^a^	23.9 ± 3.7 ^b^	20.8 ± 5.3 ^b^
Color pulp (CIELAB)				
L*	49.8 ± 4.5 ^a^	38.1 ± 1.3 ^a^	58.5 ± 2.5 ^a^	55.6 ± 4.7 ^a^
a*	12.5 ± 2.9 ^b^	11.9 ± 3.8 ^b^	−1.7 ± 0.7 ^a^	−1.7 ± 0.6 ^a^
b*	11.3 ± 6.6 ^a^	−7.5 ± 1.1 ^a^	7.3 ± 1.7 ^a^	4.9 ± 1.9 ^a^

Data expressed as mean ± standard deviation (*n* = 10). Superscript letters indicate statistically significant differences (*p* ≤ 0.05) between varieties.

**Table 2:** HPLC retention times, UV/Vis spectra, and MS spectral data of betalains and phenolic compounds in prickly pears (*Opuntia ficus-indica* L. Mill.) from the Canary Islands (Spain).

No ^1^	Rt (min)	Compound Identity	UV λ_max_ (nm)	[M+H] ^+^	MS/MS (*m*/*z*)
1	3.1	Bx-hydroxyproline (Portulacaxanthin I)	479	325.11	307.13, 220.10, 191.14
2	3.3	Bx-glycine (Portulacaxanthin III) ^a^	471	269.11	225.14, 136.06
3	3.7	Bx-unknown	475	307.08	116.07, 84.08, 76.02
4	3.9	Bx-asparagine (Vulgaxanthin III)	474	326.14	325.14, 307.13, 220.10
5	4.0	Bx-glutamine (Vulgaxanthin I) ^a^	470	340.11	308.09, 116.07, 84.04, 76.02
6	5.5	Bx-glutamic acid (Vulgaxanthin II) ^a^	474	341.10	292.20, 147.04, 72.08
7	6.1	Piscidic acid derivative	228, 275	322.21	147.04, 119.05, 107.05, 91.05
8	9.2	Bx-amino butyric acid	469	297.11	286.09, 153.04, 86.09
9	10.5	Bx-proline (Indicaxanthin) ^a^	478	309.11	263.10, 217.10, 70.06
10	14.0	Piscidic acid ^a^	232, 275	257.07	191.07, 147.04, 119.05, 107.05
11	15.7	Betanin ^a^	534	551.15	390.10, 389.10
12	20.7	Isobetanin	534	551.15	390.10, 389.10
13	27.3	Betanidin ^a^	540	389.10	345.09, 150.05
14	28.0	Gomphrenin I	536	551.15	389.10
15	32.3	Neobetanin	476	549.13	387.08
16	33.7	4-hydroxybenzoic acid derivative	274	205.05	161.06, 131.05, 115.05, 105.07
17	37.9	Bx-tryptophan	473	398.25	307.26, 219.05
18	38.7	Quercetin glycoside I (QG1)	266, 351	426.24	303.05, 191.07, 120.08
19	39.1	Quercetin glycoside II (QG2)	269, 350	653.28	303.05, 177.05
20	40.0	Isorhamnetin glucosyl-rhamnosyl-rhamnoside (IG1) ^a^	254, 354	771.23	625.18, 317.07, 85.03
21	40.4	Isorhamnetin glucosyl-rhamnosyl-pentoside (IG2) ^a^	253, 354	757.22	317.07, 167.07, 86.10
22	40.8	Isorhamnetin hexosyl-hexosyl-pentoside (IG3) ^a^	253, 354	757.22	317.06
23	41.2	Isorhamnetin glucosyl-pentoside (IG4) ^a^	254, 354	611.16	479.12, 317.07, 177.05
24	41.6	Quercetin-3-rutinoside (Rutin) ^a^	250, 343	611.23	303.05, 229.11, 137.07
25	42.5	Isorhamnetin hexosyl-rhamnoside (IG7) ^a^	255, 355	625.53	479.12, 317.07
26	43.3	Isorhamnetin glycoside	254, 353	581.15	317.07
27	44.1	Kaempferol glucosyl-rhamnoside (KG1) ^a^	262, 351	595.17	287.06
28	44.5	Isorhamnetin glucosyl-rhamnoside (IG5) ^a^	253, 354	625.18	317.07, 85.03
29	45.5	Isorhamnetin-hexosyl-pentoside (IG6) ^a^	253, 354	611.07	317.07
30	45.8	Isorhamnetin glycoside	232, 330	814.58	641.37, 317.07, 169.09
31	50.0	Isorhamnetin ^a^	370	317.07	317.07

^1^ Peak numbers are according to Figure 1. ^a^ Confirmed with semi-synthesized, purified or commercial standard.

**Table 3:** Individual betalain content (mg/100 g fresh weight) in prickly pear (*Opuntia ficus-indica* L. Mill.) peels and pulps from the Canary Islands by HPLC.

			Prickly Pear Variety			
No.	Compound	Tissue	Colorado *(Orange)*	Fresa *(Red)*	Blanco Buenavista *(White)*	Blanco Fasnia *(White)*
**Betaxanthins (mg/100 g fresh weight)**						
1	Bx-hydroxyproline (Portulacaxanthin I)	Peel	0.09 ± 0.00 ^b^	0.01 ± 0.00 ^a^	n.d. ^a^	n.d. ^a^
		Pulp	0.06 ± 0.00 ^c^	0.01 ± 0.00 ^b^	n.d. ^a^	n.d. ^a^
2	Bx-glycine (Portulacaxanthin III)	Peel	0.94 ± 0.02 ^c^	0.11 ± 0.01 ^b^	0.02 ± 0.00 ^a^	0.01 ± 0.00 ^a^
		Pulp	1.16 ± 0.02 ^c^	0.30 ± 0.01 ^b^	0.03 ± 0.00 ^a^	0.02 ± 0.00 ^a^
3	Bx-unknown	Peel	0.19 ± 0.00 ^c^	0.03 ± 0.00 ^b^	tr. ^a^	0.01 ± 0.00 ^a^
		Pulp	0.27 ± 0.00 ^d^	0.06 ± 0.00 ^c^	n.d. ^a^	0.02 ± 0.00 ^b^
4	Bx-asparagine (Vulgaxanthin III)	Peel	0.67 ± 0.01 ^c^	0.02 ± 0.00 ^b^	tr. ^a^	n.d. ^a^
		Pulp	0.24 ± 0.01 ^c^	0.08 ± 0.02 ^b^	n.d. ^a^	n.d. ^a^
5	Bx-glutamine (Vulgaxanthin I)	Peel	0.21 ± 0.00 ^c^	0.02 ± 0.00 ^b^	tr. ^a^	n.d. ^a^
		Pulp	0.29 ± 0.01 ^c^	0.06 ± 0.00 ^b^	n.d. ^a^	n.d. ^a^
6	Bx-glutamic acid (Vulgaxanthin II)	Peel	0.20 ± 0.00 ^c^	0.02 ± 0.00 ^b^	n.d. ^a^	n.d. ^a^
		Pulp	0.09 ± 0.00 ^c^	0.02 ± 0.00 ^b^	n.d. ^a^	n.d. ^a^
9	Bx-amino butyric acid	Peel	0.12 ± 0.00 ^c^	0.01 ± 0.00 ^b^	n.d. ^a^	n.d. ^a^
		Pulp	0.16 ± 0.02 ^c^	0.04 ± 0.00 ^b^	n.d. ^a^	n.d. ^a^
10	Bx-proline (Indicaxanthin)	Peel	5.98 ± 0.19 ^c^	1.24 ± 0.06 ^b^	0.03 ± 0.00 ^a^	0.02 ± 0.00 ^a^
		Pulp	11.79 ± 0.15 ^c^	4.01 ± 0.14 ^b^	0.05 ± 0.00 ^a^	0.09 ± 0.01 ^a^
18	Bx-tryptophan	Peel	0.20 ± 0.00 ^c^	0.02 ± 0.00 ^b^	0.02 ± 0.00 ^b^	n.d. ^a^
		Pulp	0.20 ± 0.00 ^c^	0.05 ± 0.00 ^b^	tr. ^a^	n.d. ^a^
**Betacyanins (mg/100 g fresh weight)**						
12	Betanin	Peel	0.54 ± 0.02 ^a^	9.54 ± 0.59 ^b^	0.02 ± 0.00 ^a^	0.01 ± 0.00 ^a^
		Pulp	0.62 ± 0.02 ^b^	11.17 ± 0.28 ^c^	0.03 ± 0.00 ^a^	0.01 ± 0.00 ^a^
13	Isobetanin	Peel	0.07 ± 0.00 ^a^	0.51 ± 0.09 ^b^	n.d. ^a^	n.d. ^a^
		Pulp	0.05 ± 0.01 ^a^	1.02 ± 0.12 ^b^	n.d. ^a^	n.d. ^a^
14	Betanidin	Peel	0.02 ± 0.00 ^a^	0.18 ± 0.01 ^b^	tr. ^a^	n.d. ^a^
		Pulp	0.02 ± 0.00 ^a^	0.33 ± 0.02 ^b^	tr. ^a^	n.d. ^a^
15	Gomphrenin I	Peel	0.02 ± 0.00 ^a^	0.31 ± 0.02 ^b^	tr. ^a^	n.d. ^a^
		Pulp	0.02 ± 0.00 ^b^	0.31 ± 0.00 ^c^	tr. ^a^	n.d. ^a^
16	Neobetanin	Peel	0.04 ± 0.00 ^b^	0.17 ± 0.00 ^c^	n.d. ^a^	n.d. ^a^
		Pulp	n.d. ^a^	tr. ^a^	n.d. ^a^	n.d. ^a^
	**Total betaxanthins ^1^**	Peel	8.61 ± 0.24 ^c^	1.48 ± 0.07 ^b^	0.07 ± 0.00 ^a^	0.04 ± 0.00 ^a^
		Pulp	14.26 ± 0.14 ^c^	4.63 ± 0.13 ^b^	0.08 ± 0.00 ^a^	0.13 ± 0.01 ^a^
	**Total betacyanins ^1^**	Peel	0.68 ± 0.03 ^a^	10.71 ± 0.72 ^b^	0.02 ± 0.00 ^a^	0.01 ± 0.00 ^a^
		Pulp	0.71 ± 0.02 ^b^	12.84 ± 0.18 ^c^	0.03 ± 0.00 ^a^	0.01 ± 0.00 ^a^
	**Total betalains ^1^**	Peel	9.29 ± 0.27 ^b^	12.19 ± 0.80 ^c^	0.09 ± 0.00 ^a^	0.05 ± 0.00 ^a^
		Pulp	14.97 ± 0.12 ^b^	17.47 ± 0.31 ^c^	0.11 ± 0.00 ^a^	0.14 ± 0.01 ^a^

Results were expressed as mean ± standard deviation (*n* = 4). This came from obtaining at least two independent extracts (*n* = 2) and performing the determinations of each two times (*n* = 2). Superscript letters indicate statistically significant differences (*p* ≤ 0.05) between varieties. ^1^ Expressed as mg/100 g fresh weight. Abbreviations: n.d.: not detected; tr.: traces.

**Table 4:** Individual phenolic acid and flavonoid content (mg/100 g fresh weight) in prickly pear (*Opuntia ficus-indica* L. Mill.) peels and pulps from the Canary Islands by HPLC.

			Prickly Pear Variety			
No.	Compound	Tissue	Colorada *(Orange)*	Fresa *(Red)*	Blanco Buenavista *(White)*	Blanco Fasnia *(White)*
**Phenolic acids (mg/100 g fresh weight)**						
7	Piscidic acid derivative	Peel	15.85 ± 0.70 ^c^	10.55 ± 0.74 ^b^	14.92 ± 0.38 ^c^	4.94 ± 0.02 ^a^
		Pulp	13.49 ± 0.17 ^b^	13.42 ± 0.69 ^b^	14.04 ± 0.40 ^b^	1.33 ± 0.11 ^a^
10	Piscidic acid	Peel	407.35 ± 13.44 ^b^	396.88 ± 9.99 ^b^	423.61 ± 37.96 ^b^	307.94 ± 1.26 ^a^
		Pulp	47.49 ± 0.05 ^b^	36.53 ± 0.20 ^a^	46.33 ± 0.58 ^b^	35.41 ± 0.55 ^a^
16	4-hydroxybenzoic acid derivative	Peel	14.62 ± 0.41 ^d^	9.17 ± 0.16 ^c^	4.88 ± 0.42 ^a^	7.10 ± 0.06 ^b^
		Pulp	1.18 ± 0.01 ^b^	0.83 ± 0.03 ^a^	1.34 ± 0.06 ^c^	1.23 ± 0.01 ^ab^
**Flavonoids (mg/100 g fresh weight)**						
18	Quercetin glycoside (QG1)	Peel	0.79 ± 0.00 ^b^	0.86 ± 0.06 ^b^	0.53 ± 0.01 ^a^	0.53 ± 0.01 ^a^
		Pulp	tr. ^a^	tr. ^a^	tr. ^a^	tr. ^a^
19	Quercetin glycoside (QG2)	Peel	0.73 ± 0.01 ^c^	0.78 ± 0.02 ^d^	0.51 ± 0.01 ^b^	0.34 ± 0.01 ^a^
		Pulp	tr. ^a^	tr. ^a^	tr. ^a^	tr. ^a^
20	Isorhamnetin glucosyl-rhamnosyl-rhamnoside (IG1)	Peel	1.80 ± 0.03 ^b^	2.17 ± 0.19 ^c^	1.11 ± 0.02 ^a^	1.40 ± 0.01 ^a^
		Pulp	0.01 ± 0.00 ^a^	0.02 ± 0.00 ^b^	0.03 ± 0.00 ^bc^	0.03 ± 0.00 ^d^
21	Isorhamnetin glucosyl-rhamnosyl-pentoside (IG2)	Peel	1.07 ± 0.03 ^b^	2.21 ± 0.21 ^c^	0.53 ± 0.01 ^a^	1.38 ± 0.01 ^b^
		Pulp	0.01 ± 0.00 ^a^	0.02 ± 0.00 ^c^	0.01 ± 0.00 ^b^	0.02 ± 0.00 ^c^
22	Isorhamnetin hexosyl-hexosyl-pentoside (IG3)	Peel	0.48 ± 0.01 ^b^	0.64 ± 0.03 ^c^	0.13 ± 0.00 ^a^	0.44 ± 0.00 ^b^
		Pulp	tr. ^a^	0.01 ± 0.00 ^b^	tr. ^b^	0.01 ± 0.00 ^c^
23	Isorhamnetin glucosyl-pentoside (IG4)	Peel	0.95 ± 0.01 ^c^	1.38 ± 0.06 ^d^	0.16 ± 0.05 ^a^	0.72 ± 0.04 ^b^
		Pulp	tr. ^a^	0.01 ± 0.00 ^a^	0.01 ±0.00 ^a^	0.01 ± 0.00 ^b^
24	Quercetin-3-rutinoside (Rutin)	Peel	1.33 ± 0.19 ^b^	1.22 ± 0.14 ^b^	0.45 ± 0.04 ^a^	0.75 ± 0.01 ^a^
		Pulp	tr. ^a^	tr. ^a^	0.01 ± 0.00 ^a^	0.03 ± 0.00 ^b^
25	Isorhamnetin hexosyl-rhamnoside (IG7)	Peel	n.d. ^a^	n.d. ^a^	1.25 ± 0.07 ^a^	n.d. ^a^
		Pulp	n.d. ^a^	n.d. ^a^	0.01 ± 0.00 ^a^	n.d. ^a^
26	Isorhamnetin glycoside	Peel	tr. ^a^	0.07 ± 0.01 ^b^	0.14 ± 0.02 ^c^	n.d. ^a^
		Pulp	tr. ^a^	tr. ^a^	tr. ^a^	n.d. ^a^
27	Kaempferol glucosyl-rhamnoside (KG1)	Peel	0.27 ± 0.04 ^a^	0.36 ± 0.00 ^a^	0.94 ± 0.19 ^b^	0.15 ± 0.01 ^a^
		Pulp	0.01 ± 0.00 ^b^	0.02 ± 0.00 ^b^	0.08 ± 0.00 ^c^	0.01 ± 0.00 ^a^
28	Isorhamnetin glucosyl-rhamnoside (IG5)	Peel	2.85 ± 0.07^c^	3.21 ± 0.07 ^d^	1.15 ± 0.05 ^a^	2.03 ± 0.00 ^b^
		Pulp	0.01 ± 0.00 ^a^	0.02 ± 0.00 ^b^	0.05 ± 0.00 ^d^	0.03 ± 0.00 ^c^
29	Isorhamnetin hexosyl-pentoside (IG6)	Peel	0.37 ± 0.06 ^c^	0.23 ± 0.01 ^b^	0.45 ± 0.01 ^c^	0.12 ± 0.01 ^a^
		Pulp	tr. ^a^	0.01 ± 0.00 ^a^	tr. ^a^	n.d. ^a^
30	Isorhamnetin glycoside	Peel	n.d. ^a^	n.d. ^a^	0.37 ± 0.03 ^a^	n.d. ^a^
		Pulp	n.d. ^a^	n.d. ^a^	tr. ^a^	n.d. ^a^
31	Isorhamnetin	Peel	0.33 ± 0.01 ^b^	0.62 ± 0.03 ^d^	0.53 ± 0.02 ^c^	n.d. ^a^
		Pulp	tr. ^a^	tr. ^a^	tr. ^a^	tr. ^a^
	**Total phenolic acids ^1^**	Peel	437.82 ± 14.55 ^b^	416.60 ± 11.65 ^b^	443.41 ± 38.41 ^b^	319.98 ± 13.01 ^a^
		Pulp	62.16 ± 0.21 ^c^	50.78 ± 0.46 ^b^	61.71 ± 0.93 ^c^	37.98 ± 0.67 ^a^
	**Total flavonoids ^1^**	Peel	11.49 ± 0.17 ^b^	13.76 ± 0.75 ^b^	8.25 ± 0.40 ^a^	7.84 ± 0.07 ^a^
		Pulp	0.04 ± 0.00 ^a^	0.09 ± 0.01 ^c^	0.19 ± 0.01 ^d^	0.14 ± 0.01 ^c^
	**Total phenolic compounds ^1^**	Peel	449.31 ± 14.38 ^a^	430.36 ± 11.65 ^a^	451.66 ± 38.02 ^a^	327.82 ± 1.37 ^b^
		Pulp	62.20 ± 0.21 ^c^	50.87 ± 0.45 ^b^	61.90 ± 0.93 ^c^	38.12 ± 0.68 ^a^

Results were expressed as mean ± standard deviation (*n* = 4). This came from obtaining at least two independent extracts (*n* = 2) and performing the determinations of each two times (*n* = 2). Superscript letters indicate statistically significant differences (*p* ≤ 0.05) between varieties. ^1^ Expressed as mg/100 g fresh weight. Abbreviations: n.d.: not detected; tr.: traces.

**Table 5:** Bioaccessibility (%) of betalains and phenolic compounds in prickly pear pulps after simulated in vitro gastrointestinal digestion.

Compound Identity	Colorado *(Orange)*	Fresa *(Red)*	Blanco Buenavista *(White)*	Blanco Fasnia *(White)*
Indicaxanthin	58.2 ± 5.0 ^b^	68.9 ± 5.1 ^b^	n.d. ^a^	n.d. ^a^
Betanin	46.2 ± 1.1 ^b^	45.6 ± 5.9 ^b^	n.d. ^a^	n.d. ^a^
Piscidic acid	46.7 ± 4.2 ^b^	38.6 ± 6.9 ^b^	27.4 ± 2.7 ^a^	47.1 ± 4.8 ^b^
4-hydroxybenzoic acid derivative	16.6 ± 3.1 ^b^	23.4 ± 1.3 ^c^	9.6 ± 3.1 ^a^	17.8 ± 1.0 ^b^

Results were expressed as mean ± standard deviation (*n* = 4). This came from obtaining at least two independent digestions (*n* = 2) and performing the determinations of each two times (*n* = 2). Superscript letters indicate statistically significant differences (*p* ≤ 0.05) between varieties. Abbreviations: n.d.: not detected.

**Table 6:** Bioaccessibility (%) of betalains and phenolic compounds in prickly pear peels after simulated in vitro gastrointestinal digestion.

Compound Identity	Colorado *(Orange)*	Fresa *(Red)*	Blanco Buenavista *(White)*	Blanco Fasnia *(White)*
Indicaxanthin	68.2 ± 4.0 ^b^	70.4 ± 4.5 ^b^	n.d. ^a^	n.d. ^a^
Betanin	54.1 ± 1.1 ^c^	38.6 ± 0.8 ^b^	n.d. ^a^	n.d. ^a^
Piscidic acid	51.8 ± 0.4 ^a^	54.8 ± 5.1 ^a^	79.9 ± 2.3 ^b^	69.4 ± 4.6 ^b^
4-hydroxybenzoic acid derivative	52.3 ± 0.9 ^a^	65.8 ± 3.4 ^b^	68.9 ± 0.9 ^b^	77.1 ± 5.0 ^b^
Isorhamnetin glucosyl-rhamnosyl-rhamnoside (IG1)	45.5 ± 2.2 ^a^	51.6 ± 9.2 ^a^	54.1 ± 11.0 ^a^	64.2 ± 3.5 ^a^
Isorhamnetin glucosyl-rhamnosyl-pentoside (IG2)	52.6 ± 1.0 ^a^	51.1 ± 6.7 ^a^	60.9 ± 14.8 ^a^	70.6 ± 4.1 ^a^
Isorhamnetin hexosyl-hexosyl-pentoside (IG3)	51.0 ± 5.1 ^b^	55.3 ± 8.8 ^b^	n.d. ^a^	63.6 ± 3.2 ^b^
Isorhamnetin glucosyl-pentoside (IG4)	50.2 ± 7.8 ^a^	50.6 ± 6.0 ^a^	82.5 ± 15.2 ^a^	72.1 ± 6.1 ^a^
Isorhamnetin hexosyl-rhamnoside (IG7)	n.a. ^a^	n.a. ^a^	29.5 ± 9.5 ^b^	n.a. ^a^
Isorhamnetin glucosyl-rhamnoside (IG5)	32.1 ± 2.8 ^a^	32.4 ± 2.2 ^a^	25.9 ± 5.9 ^a^	63.2 ± 4.7 ^b^

Results were expressed as mean ± standard deviation (*n* = 4). This came from obtaining at least two independent digestions (*n* = 2) and performing the determinations of each two times *(n* = 2). Superscript letters indicate statistically significant differences (*p* ≤ 0.05) between varieties. Abbreviations: n.d.: not detected; n.a.: not applicable.

## Supplementary Materials

The following are available online at https://www.mdpi.com/2076-3921/9/2/164/s1, Table S1: Bioactive content (mg/100 g fresh weight) in prickly pear pulps during in vitro simulated gastrointestinal digestion, Table S2: Bioactive content (mg/100 g fresh weight) in prickly pear peels during in vitro simulated gastrointestinal digestion.
