# Supplementary material for: Digestive Stability and Bioaccessibility of Antioxidants in Prickly Pear Fruits from the Canary Islands: Healthy Foods and Ingredients
Source: Antioxidants (Basel). 2020 Feb 17;9(2):164. doi: 10.3390/antiox9020164 (PMC7070381; doi:10.3390/antiox9020164)
Supplement: Supplementary file 1 [file antioxidants-09-00164-s001.zip › antioxidants-720346_Supplementary Table S1_R1.pdf]

Supplementary Table S1. Bioactive content (mg/100g fresh weight) in prickly pear pulps during *in vitro* simulated gastrointestinal digestion.

	**Colorada**				**Fresa**			
**Compounds**	**Fruit**	**Oral**	**Gastric**	**Intestinal**	**Fruit**	**Oral**	**Gastric**	**Intestinal**
Indicaxanthin	11.79 ± 0.15 ^{c}	11.54 ± 0.03 ^{c}	8.67 ± 0.05 ^{b}	6.87 ± 0.19 ^{a}	4.01 ± 0.14 ^{b}	4.12 ± 0.15 ^{b}	4.01 ± 0.35 ^{b}	2.76 ± 0.07 ^{a}
Betanin	0.62 ± 0.02 ^{c}	0.34 ± 0.00 ^{b}	0.49 ± 0.07 ^{b}	0.28 ± 0.02 ^{a}	11.17 ± 0.28 ^{c}	12.32 ± 0.35 ^{c}	9.36 ± 0.74 ^{b}	5.09 ± 0.48 ^{a}
Piscidic acid	47.49 ± 0.05 ^{b}	25.21 ± 1.49 ^{a}	26.76 ± 0.48 ^{a}	22.19 ± 2.05 ^{a}	36.53 ± 0.20 ^{c}	30.18 ± 0.04 ^{b}	27.24 ± 2.06 ^{b}	14.08 ± 2.41 ^{a}
4-hydroxybenzoic acid derivative	1.18 ± 0.01 ^{b}	1.00 ± 0.02 ^{b}	0.25 ± 0.04 ^{a}	0.20 ± 0.04 ^{a}	0.83 ± 0.03 ^{d}	0.70 ± 0.06 ^{c}	0.32 ± 0.03 ^{b}	0.19 ± 0.02 ^{a}
	**Blanco Buenavista**				**Blanco Fasnia**			
**Compounds**	**Fruit**	**Oral**	**Gastric**	**Intestinal**	**Fruit**	**Oral**	**Gastric**	**Intestinal**
Indicaxanthin	0.05 ± 0.00 ^{b}	0.03 ± 0.00 ^{ab}	0.03 ± 0.01 ^{ab}	0.02 ± 0.00 ^{a}	0.09 ± 0.01 ^{c}	0.04 ± 0.01 ^{b}	0.06 ± 0.00 ^{bc}	0.03 ± 0.00^{a}
Betanin	0.03 ± 0.01 ^{b}	0.03 ± 0.01 ^{b}	0.01 ± 0.00 ^{b}	n.d. ^{a}	0.01 ± 0.00 ^{b}	n.d. ^{a}	n.d. ^{a}	n.d. ^{a}
Piscidic acid	44.33 ± 0.58 ^{c}	29.25 ± 2.00 ^{b}	29.04 ± 1.64 ^{b}	12.70 ± 0.33 ^{a}	35.41 ± 0.55 ^{d}	29.42 ± 1.02 ^{c}	26.02 ± 1.36 ^{b}	16.68 ± 1.00 ^{a}
4-hydroxybenzoic acid derivative	1.34 ± 0.06 ^{b}	1.25 ± 0.03 ^{b}	0.18 ± 0.02 ^{a}	0.13 ± 0.03 ^{a}	1.23 ± 0.01 ^{c}	1.15 ± 0.01 ^{c}	0.44 ± 0.10 ^{b}	0.22 ± 0.03 ^{a}

Results were expressed as mean ± standard deviation (n=4). This came from obtaining at least two independent digestions (n=2) and performing the determinations of each two times (n=2). Superscript letters indicate statistically significant differences ($p \leq 0.05$) between the fruit and the digestive stages for each variety and compound. Abbreviations: n.d.: not detected.
