# Supplementary material for: Digestive Stability and Bioaccessibility of Antioxidants in Prickly Pear Fruits from the Canary Islands: Healthy Foods and Ingredients
Source: Antioxidants (Basel). 2020 Feb 17;9(2):164. doi: 10.3390/antiox9020164 (PMC7070381; doi:10.3390/antiox9020164)
Supplement: Supplementary file 1 [file antioxidants-09-00164-s001.zip › antioxidants-720346_Supplementary Table S2_R1.pdf]

Supplementary Table S2. Bioactive content (mg/100g fresh weight) in prickly pear peels during in vitro simulated gastrointestinal digestion.

	**Colorada**				**Fresa**			
**Compounds**	**Fruit**	**Oral**	**Gastric**	**Intestinal**	**Fruit**	**Oral**	**Gastric**	**Intestinal**
Indicaxanthin	5.98 ± 0.19 ^{d}	6.10 ± 0.03 ^{c}	5.59 ± 0.09 ^{b}	4.08 ± 0.11 ^{a}	1.24 ± 0.06 ^{c}	1.20 ± 0.01 ^{b}	1.31 ± 0.07 ^{b}	0.87 ± 0.00 ^{a}
Betanin	0.54 ± 0.02 ^{b}	0.48 ± 0.07 ^{b}	0.54 ± 0.03 ^{b}	0.29 ± 0.03 ^{a}	9.54 ± 0.59 ^{b}	9.94 ± 1.09 ^{b}	8.53 ± 0.09 ^{b}	3.68 ± 0.22 ^{a}
Piscidic acid	407.35 ± 13.44 ^{b}	430.07 ± 11.65 ^{c}	452.90 ± 5.59 ^{c}	221.21 ± 8.07 ^{a}	396.88 ± 9.99 ^{b}	442.99 ± 22.84 ^{b}	451.17 ± 20.32 ^{b}	217.64 ± 21.90 ^{a}
4-hydroxybenzoic acid derivative	14.62 ± 0.41 ^{b}	15.01 ± 0.33 ^{b}	16.02 ± 0.32 ^{b}	7.49 ± 0.11 ^{a}	9.17 ± 0.16 ^{b}	11.33 ± 1.19 ^{b}	11.48 ± 0.44 ^{b}	6.04 ± 0.31 ^{a}
IG1	1.80 ± 0.03 ^{b}	1.89 ± 0.05 ^{b}	1.92 ± 0.02 ^{b}	0.82 ± 0.08 ^{a}	2.17 ± 0.19 ^{b}	2.24 ± 0.04 ^{b}	2.23 ± 0.27 ^{b}	1.12 ± 0.15 ^{a}
IG2	1.61 ± 0.03 ^{b}	1.85 ± 0.04 ^{c}	1.82 ±0.27 ^{c}	0.84 ± 0.00 ^{a}	2.21 ± 0.21 ^{b}	2.26 ± 0.10 ^{b}	2.43 ± 0.03 ^{b}	1.13 ± 0.06 ^{a}
IG3	0.48 ± 0.01 ^{b}	0.50 ± 0.03 ^{b}	0.59 ± 0.18 ^{b}	0.24 ±0.03 ^{a}	0.64 ± 0.03 ^{b}	0.71 ± 0.12 ^{b}	0.68 ± 0.05 ^{b}	0.35 ± 0.14 ^{a}
IG4	0.95 ± 0.01 ^{b}	1.04 ± 0.03 ^{b}	1.10 ± 0.00 ^{b}	0.47 ± 0.11 ^{a}	1.38 ± 0.06 ^{b}	1.33 ± 0.02 ^{b}	1.36 ± 0.15 ^{b}	0.70 ± 0.08 ^{a}
IG7	n.d. ^{a}	n.d. ^{a}	n.d. ^{a}	n.d. ^{a}	n.d. ^{a}	n.d. ^{a}	n.d. ^{a}	n.d. ^{a}
IG5	2.85 ± 0.07 ^{c}	2.30 ± 0.02 ^{b}	1.94 ± 0.40 ^{b}	0.91 ± 0.16 ^{a}	3.21 ± 0.07 ^{d}	2.39 ± 0.02 ^{c}	1.96 ± 0.00 ^{b}	1.04 ± 0.07 ^{a}
	**Blanco Buenavista**				**Blanco Fasnia**			
**Compounds**	**Fruit**	**Oral**	**Gastric**	**Intestinal**	**Fruit**	**Oral**	**Gastric**	**Intestinal**
Indicaxanthin	0.03 ± 0.00 ^{b}	0.02 ± 0.00 ^{b}	n.d. ^{a}	n.d. ^{a}	0.02 ± 0.00 ^{ab}	0.02 ± 0.00 ^{ab}	n.d. ^{a}	n.d. ^{a}
Betanin	0.02 ± 0.00 ^{b}	n.d. ^{a}	n.d. ^{a}	n.d. ^{a}	0.01 ± 0.00 ^{b}	n.d. ^{a}	n.d. ^{a}	n.d. ^{a}
Piscidic acid	423.61 ± 37.96 ^{b}	410.67 ± 24.88 ^{b}	400.65 ± 29.39 ^{b}	338.46 ± 30.83 ^{a}	307.95 ± 1.26 ^{b}	320.76 ± 36.37 ^{b}	315.26 ± 78.06 ^{b}	213.59 ± 19.83 ^{a}
4-hydroxybenzoic acid derivative	4.88 ± 0.42 ^{b}	3.76 ± 0.28 ^{a}	3.53 ± 0.33 ^{a}	3.36 ± 0.50 ^{a}	7.10 ± 0.06 ^{b}	8.53 ± 0.90 ^{b}	8.41 ± 2.17 ^{b}	5.47 ± 0.46 ^{a}
IG1	1.11 ± 0.02 ^{b}	0.72 ± 0.05 ^{a}	0.78 ± 0.06 ^{a}	0.60 ± 0.20 ^{a}	1.40 ± 0.01 ^{b}	1.44 ± 0.15 ^{b}	1.36 ± 0.39 ^{b}	0.90 ± 0.06 ^{a}
IG2	0.53 ± 0.01 ^{b}	0.39 ± 0.03 ^{a}	0.41 ± 0.03 ^{a}	0.32 ± 0.11 ^{a}	1.38 ± 0.01 ^{b}	1.42 ± 0.15 ^{b}	1.31 ± 0.17 ^{b}	0.97 ± 0.07 ^{a}
IG3	0.13 ± 0.00 ^{b}	0.05 ± 0.00 ^{a}	n.d. ^{a}	n.d. ^{a}	0.44 ± 0.00 ^{b}	0.51 ± 0.04 ^{b}	0.49 ± 0.03 ^{b}	0.28 ± 0.02 ^{a}
IG4	0.16 ± 0.05 ^{a}	0.17 ± 0.01 ^{a}	0.33 ± 0.01 ^{b}	0.13 ± 0.03 ^{a}	0.72 ± 0.04 ^{b}	0.81 ± 0.07 ^{b}	0.72 ± 0.21 ^{b}	0.52 ± 0.02 ^{a}
IG7	1.25 ± 0.07 ^{c}	1.15 ± 0.05 ^{c}	0.70 ± 0.06 ^{b}	0.37 ± 0.15 ^{a}	n.d. ^{a}	n.d. ^{a}	n.d. ^{a}	n.d. ^{a}
IG5	1.15 ± 0.05 ^{c}	0.52 ± 0.04 ^{b}	0.54 ± 0.02 ^{b}	0.30 ± 0.08 ^{a}	2.03 ± 0.00 ^{b}	2.30 ± 0.17 ^{b}	2.10 ± 0.24 ^{b}	1.28 ± 0.14 ^{a}

Results were expressed as mean ± standard deviation (*n*=4). This came from obtaining at least two independent digestions (*n*=2) and performing the determinations of each two times (*n*=2). Superscript letters indicate statistically significant differences ($p \leq 0.05$) between the fruit and the digestive stages for each variety and compound. Abbreviations: n.d.: not detected.
